# Ethical, Regulatory and Market related aspects of Deploying Triple Artemisinin-Based Combination Therapies for Malaria treatment in Africa: A study protocol.

**DOI:** 10.12688/wellcomeopenres.16065.1

**Published:** 2021-04-07

**Authors:** Paulina Tindana, Freek de Haan, Olugbenga Ayodeji Mokuolu, Rosemonde Guissou, Oladimeji Akeem Bolarinwa, Jean Bosco Ouedraogo, Fatoumata Tou, Wouter P.C Boon, Ellen H.M Moors, Arjen M Dondorp, Mehul Dhorda, Chanaki Amaratunga, Phaik Yeong Cheah

**Affiliations:** 1 Department of Health Policy, Planning and Management, School of Public Health, College of Health Sciences, University of Ghana, Accra, Ghana; 2 Copernicus Institute of Sustainable Development, Utrecht University, Ultrecht, The Netherlands; 3 Paediatrics Department, University of Ilorin, Illorin, Nigeria; 4 Institut de Recherche en Sciences de la Sante, Bobo-Dioulasso, Burkina Faso; 5 Department of Epidemiology and Community Health, University of Illorin, Illorin, Nigeria; 6 Institut des Sciences et Techniques, Bobo-Dioulasso, Burkina Faso; 7 Mahidol-Oxford Tropical Medicine Research Unit, Faculty of Tropical Medicine, Mahidol University, Bangkok, Thailand; 8 Center for Tropical Medicine and Global Health, Nuffield Department of Medicine, University of Oxford, Oxford, UK

**Keywords:** Malaria, Africa, drug resistance, ethics

## Abstract

**Introduction: **According to the World Malaria Report 2019, Africa accounts for 94% of the global malaria deaths. While malaria prevalence and mortality have declined over the years, recent reports suggest that these gains may stand the risk of being reversed if resistance to Artemisinin Combination Therapies (ACTs) spreads from Southeast Asia to Africa. Efforts are being made to develop new treatments that will address the looming threat of ACT resistance, including the development of triple artemisinin combination therapies (TACTs). The proposed study seeks to explore the views of stakeholders on the key ethical, regulatory and market-related issues that should be considered in the potential introduction of triple artemisinin combination therapies (TACTs) in Africa.

**Methods: **The study employed qualitative research methods involving in-depth interviews and focus group discussions (FGDs) with stakeholders, who will be directly affected by the potential deployment of triple artemisinin combination treatments, as regulators, suppliers and end-users. Participants will be purposively selected and will include national regulatory authorities, national malaria control programs, clinicians, distributors and retailers as well as community members in selected districts in Burkina Faso and Nigeria.

**Discussion: **The proposed study is unique in being one of the first studies that seeks to understand the ethical, social, regulatory and market position issues prior to the development of a prospective antimalarial medicine.

## Introduction

In the early 2000s, following widespread development of antimalarial drug resistance to hitherto effective monotherapies, such as chloroquine and sulphadoxine-pyrimethamine (SP), the World Health Organization (WHO) recommended artemisinin-based combination therapies (ACTs) as an approach to improve therapeutic efficacy and delay the development of antimalarial drug resistance ( WHO, 2003). Subsequently, several malaria endemic countries in Asia and Africa changed their national malaria treatment policies to ACTs as first-line treatment for uncomplicated malaria ( Kamya *et al.,* 2002; Mulligan *et al.,* 2006; Williams *et al.,* 2004).

Artemether-lumefantrine (AL) and artesunate-amodiaquine (ASAQ) are currently the most deployed ACT combinations used by national malaria control programs in Africa and the recent World Malaria Report suggests that the efficacy rates of these ACTs ‘were more than 98%, and efficacy has remained high over time’ ( WHO, 2019). The report also showed that in 2018, there were an estimated 405,000 deaths from malaria globally, compared with 416,000 estimated deaths in 2017, and 585,000 in 2010 ( WHO, 2019). Majority (94%) of these deaths occurred in the African region ( WHO, 2019). While malaria prevalence and mortality has declined over the years, recent reports suggest that these gains may stand the risk of being reversed, particularly with the threat of resistance to ACTs spreading from Southeast Asia to Africa ( Ashley *et al.,* 2014). There is therefore the need to intensify efforts at developing effective treatment regimens that can treat multi-drug resistant *Plasmodium falciparum* malaria.

The Tracking Resistance to Artemisinin Collaboration II (TRACII) trial was established to conduct a randomized clinical trial mainly in Asia to compare the efficacy, safety and tolerability of two triple ACTs (TACTs) with corresponding ACT ( van der Pluijm *et al.*, 2020). This TRACII trial involved combining existing antimalarial: AL with amodiaquine and dihydroartemisinin-piperaquine (DHA-PPQ) with mefloquine, both of which were able to treat multi-drug resistant falciparum malaria. Taking the results to the next level, the overarching goal of the Development of Triple Artemisinin-based Combination Therapies (DeTACT) project is to have co-formulated TACTs ready for global deployment once they are found to be non-inferior to ACTs in the treatment of malaria. This has implications. As with the introduction of any new health interventions, there is the need, not only to address issues related to efficacy and safety, but also to anticipate ethical, social and regulatory aspects of deploying TACTs. Ethical questions may arise around deployment of TACTs in contexts where ACTs remain effective, such as in Africa. Effective implementation of updated treatment policies and integrating ACTs into established market structures in Africa has historically faced several ethical, social and regulatory challenges ( Maslove *et al.,* 2009; Mutero *et al.,* 2014; Wasunna *et al.,* 2008; Yeung & White, 2005). A review article by Amin *et al.* reported that it took nearly 3 years for Kenya to implement ACT after the policy change was announced ( Amin *et al.,* 2007). Similar implementation delays were observed by Bosman and Mendis in several malaria endemic countries ( Bosman & Mendis, 2007). Beyond delays in implementation programs, many factors were associated with a problematic market introduction of ACTs in Africa. These vary from high consumer prices and suboptimal integration in distribution chains, to a lack of end-user acceptability through factors such as negative community perceptions and perceived side effects ( Arrow *et al.,* 2004; Malik *et al.,* 2006; Martins *et al.,* 2012; Palafox *et al.,* 2014; Yadav *et al.,* 2007). Similar challenges are also likely to affect the future deployment of new antimalarials on the African continent. Additionally, introduction of previous generations of antimalarial drugs warrant a market positioning assessment to address issues related to changing treatment policy and the integration of TACTs into established market structures. Therefore, it is important to learn lessons from the past and anticipate ethical, regulatory and market related issues for the introduction and deployment of new generations of antimalarials.

There are several lessons that can be learned from country experiences in the adoption and deployment of ACTs in Africa which can inform the rapid deployment of TACTs in Africa. Developing effective communication strategies to sensitize communities about the change in treatment policies is clearly important and has played a major role in malaria treatment policy implementation in several countries ( Mutero *et al.,* 2014). Public involvement (which includes taking into account stakeholders’ views needs to be harnessed right from the time of undertaking the research through to implementing the national treatment policies. Innovative drugs should also be aligned with the prevalent institutional frameworks and there should be effective strategies in place to phase out previous drug generations from the market ( Martins *et al.,* 2012). Even though the WHO recommended the deployment of ACTs in areas of drug resistance since 2001, non-artemisinin therapies continued to dominate the drug markets in African countries beyond 2009 ( Palafox *et al.,* 2014). Beyond influencing policy change, there are other barriers to effective treatment of malaria that need to be addressed. Chuma *et al.* have reported that issues related to affordability, acceptability and availability interact to influence access to prompt and effective treatment ( Chuma *et al.,* 2010). Other issues that have been highlighted in the literature include community acceptance, adherence to treatment guidelines both at the facility and household level, prescribers’ preferences and the burden on health systems ( Ajayi *et al.,* 2008; Bosman & Mendis, 2007; O’Connell *et al.,* 2011). Inertia in the process of policy implementation, misalignment with institutional arrangements and complexity of distribution chains have also been associated with the slow uptake of innovative drugs ( Martins *et al.,* 2012; Palafox *et al.,* 2014; Yadav *et al.,* 2007). Ajayi *et al.,* 2008; Williams *et al.,* 2004). Addressing these issues is important to determine if TACTs will be accepted once they are proven safe and non-inferior to ACTs in African settings.

### Study protocol

The proposed study poses some uniqueness in being one of the first studies that will seek to understand the ethical, social, regulatory and market position issues prior to the development of a potential antimalarial medicine in Africa. This will inform the design, the sampling process, the tools and their validation. This paper presents the study protocol to guide the reproducibility of similar studies by other researchers and facilitate comparison of data that may be generated from such studies. 

### Research questions

Two major questions were raised for this study. The first is the ethics question around the deployment of TACTs “what are the main ethical considerations for requesting populations and governments in African countries to switch to TACTs while ACTs are still effective?” The second is the market positioning question “To what extent are the health systems and markets of African countries ready for a transition to TACTs if it is proven to be non-inferior to ACTs?”

### Aims and objectives

This study aims to explore a range of issues beyond the clinical trial results around the potential future deployment of TACTs in the current scientific, economic and social contexts, in order to prepare their potential deployment in Asia and Africa.

Specifically, the project will (i) examine the feasibility of deployment of TACTs, potential barriers to its deployment, to assess whether mitigation of observed barriers is indicated, and how the identified barriers can feed into communication plans; (ii) identify the key ethical, social, practical, and regulatory issues of changing first-line treatment from ACTs to TACTs; (iii) explore possible strategies to facilitate the integration of TACT in public and private sector distribution chains; (iv) explore the readiness of public and private sector markets for a transition to TACTs and (v) explore end-user acceptability of TACTs.

## Methods

### Study design

The study is based on a social science methodology using in-depth interviews and focus group discussions (FGDs) with key stakeholders ( Creswell, 2003). The rationale for using these qualitative methods is to allow respondents to express their views on what they value as the key considerations that researchers and national regulatory authorities should focus on for a potential deployment of TACTs in Africa.

***Context and settings.*** The project is under the auspices of the DeTACT project with the case studies conducted in Burkina Faso and Nigeria in West Africa. Both countries have high malaria prevalence and are situated on the African continent, where ACTs are still effective. According to the 2019 World Malaria Report, Nigeria accounted ‘for almost 24% of all global malaria deaths’ ( WHO, 2019). While TACTs may have limited added benefit to individual patients who could have received an ACT in the absence of artemisinin resistance in Africa; it may have a much larger benefit on the community by preventing or delaying the development of artemisinin-resistance. Moreover, health systems and malaria control policies vary significantly between both Nigeria and Burkina Faso, making both countries suitable for this multi-country assessment of the ethical and market related aspects of prospective deployment of TACTs.

### Study participants

Key stakeholders are selected from the two target countries; Burkina Faso and Nigeria and include representatives of national malaria control programs and regulatory agencies, public and private sector drug distributors and retailers, clinicians and prescribers. Finally, malaria researchers, parents, caregivers, and community leaders will be included in data collection.

### Inclusion criteria

The inclusion criteria will be male or female, aged 18 years or above and the willingness of the participant to give informed consent for participation in the study.

### Exclusion criteria

The participant may not be recruited into the study if they have been identified with intellectual and/or cognitive disability which may affect his/her ability to comprehend the information about the project.

### Study respondents

***Selection of respondents.*** At the time of writing this manuscript, data collection was completed in Nigeria while the process was on hold in Burkina Faso following the COVID-19 pandemic. Key stakeholders were approached at different levels of the healthcare system and the antimalarial drug value chain within both target countries. Initial consultations were held with the country-specific principal investigators of the DeTACT project to identify specific districts and study sites with potential participants for interviews at each of the project sites. The national regulatory authorities were contacted via telephone and personally by the country teams in Nigeria and Burkina Faso.

Five categories of respondents were purposively sampled for interviews at each selected country, as outlined in Table 1 and Table 2. The total number of interviews will depend on when we reach data saturation, when no new ideas are emerging from the interviews. Measures were put in place to ensure heterogeneity within each category of data collection. Moreover, parents who were interviewed will not be included in the DeTACT clinical trials as their perception might be biased through information.

**Table 1.** Sample for semi-structured interviews and focus group discussions (FGDs) in Nigeria.

Nigeria	Respondent	Number	Interview/FDG
Policy	National regulatory authorities	2	Interview
	National Malaria Control Program	2	Interview
Distributor	Public sector drug wholesalers/distributors	6	Interview
	Private sector wholesalers/traders	6	Interview
Health service providers	Public sector: clinicians, pharmacist	4	Interview
	Private sector: clinicians, nurses, pharmacist, drug store	4	Interview
	Village health workers	3	FGD
Researchers	Malaria researchers	5	Interview
End users	Parents /caregivers	3	FGD
	Parents/caregivers	4	Interview
	Community leaders	3	FGD

Note: FGDs will be made up of a group of 8 to 10 people

**Table 2.** Sample for semi-structured interviews and focus group discussions (FGDs) in Burkina Faso.

Burkina Faso	Respondent	Number	Interview/FDG
Policy	National regulatory authorities	2	Interview
	National Malaria Control Program	3	Interview
	Regional health director	2	Interview
Distributor	Public sector drug wholesalers/distributors	4	Interview
	Private sector wholesalers/traders	2	Interview
Health service providers	Public sector: clinicians, nurses, pharmacist	8	Interview
	Private sector: clinicians, nurses, pharmacist, informal (drug) store	8	Interview
	Village health workers	2	FGD
Researchers	Malaria researchers	6	Interview
End users	Parents /care-givers	1(m),2(f)	FGD
	Community youth group / Grin	2	FGD
	Community leaders	2	Interview

Note: FGDs will be made up of a group of 8 to 10 people.

***Development and validation of data collection tools.*** With the identification of the various stakeholders and key issues to be addressed by the different groups, draft tools for the in-depth interviews and FGDs as may be applicable to different respondents were developed. These were reviewed and validated by all the core team members, particularly the lead co-investigators from the study countries. These formed the generic data tools. Furthermore, prior to the study,, the tools were pre-tested in each of the study countries. This was to allow engaged research assistants to be trained, allow for local tweaking of the tools on how best the designed concepts can be communicated and align the tools properly to the local context of the target group. For stakeholders at the community levels that cannot communicate effectively in English, the relevant tools were translated to the local language by language experts and back-translated to English to ensure consistency of the translation.

### Data collection

Qualitative data were collected from two main sources: semi-structured interviews and FGDs.

***Semi-structured interviews.*** A semi-structured interview guide was developed to cover topics related to current treatment guidelines, prescription preferences etc. The target groups for these semi-structured interviews are regulatory authorities, National Malaria Control Programs (NMCPs), wholesalers, distributors, prescribers, clinicians, malaria researchers and community members. Most of the interviews conducted so far lasted approximately 30–45 minutes and were conducted in a quiet place at the interviewee’s request. Interviews were audio recorded, transcribed verbatim into English. If more time is required, the lead interviewer will ask the interviewee if he or she is willing to be interviewed for an additional 15 minutes; the time will only be extended if they agree. The investigator or study team member also recorded simultaneous written notes in English, which will serve as a critical back-up in case of audio recording failures. Recordings and transcripts are currently stored on a password-protected computer, and participants’ identities will remain confidential in any reports of the data. Both verbatim translation and structure transcription of the interview will be conducted by a trained transcriber who is also exposed to research methodology and the principles of confidentiality. The transcribed notes will be shared to study team members only through very secured platforms and not through surface or electronic mail or any means that can compromise the confidentiality of the data.

***FGDs.*** We identified different groups of respondents including village health workers, parents, community advisory boards and community leaders at each of the selected countries for FGDs. The FGDs were made up of a group of 8 to 10 people who are brought together to discuss an issue of interest and lasted approximately 40 to 60 minutes. The technique is useful for gathering information about the way people behave and motivations that underlie these behaviours as the discussions allow for the expression of views, opinions and counter-opinions on attitudes, beliefs and practices. Our aim in these FGDs was to explore the collective views of these respondents on what issues need to be anticipated in the deployment of TACTs in Africa, specifically on issues related to health-seeking behaviour, community acceptance and adherence to treatment guidelines at the individual, household and health facility levels. Contents of implementation programs and user preferences were also discussed in the FGDs. The recorded and transcribed information will be managed as described for the semi-structured interviews.

### Data management

***Access to data.*** Direct access will be granted to authorised representatives from the sponsor, host institutions, research ethics committees, and regulatory authorities to ensure compliance with any existing regulations.

***Data recording and record keeping.*** To facilitate the management and analysis of the data, all the interviews were audio recorded and transcribed, where interviewees consent to the use of recorders. Where permission to record is not granted, notes were taken during the interview and an account of the interview were prepared immediately afterwards. Interviews conducted in the local languages of the research participants and French, in the case of Burkina Faso, are being translated into English by the local researchers or trained translators.

### Data analysis

Data analysis will be an on-going process. We will follow the steps used across many different qualitative analytical traditions with a focus on thematic content analysis. This analysis will focus on an in-depth description and conceptual interpretation of the entire data set ( Boyatzis, 1998; Fereday & Mair-Cochrane, 2006). The analysis will initially be based on the themes used to guide data collection, and then augmented by themes emerging from the data. Qualitative analysis software NVivo version 12 ( QSR NVivo) will be used to aid in the management and analysis of the data.

### Ethical and regulatory considerations

The main ethical issues in this study relate to privacy and confidentiality. Care was taken to maintain privacy during the audio recording of interviews and interactions with individual participants. The study will comply with the General Data Protection Regulation (GDPR), which requires that personal data must not be kept as identifiable data for longer than necessary for the purposes concerned. All completed interview transcripts, field notes, etc. will be de-identified and stored in a secure and access restricted place at the collection site on password secured servers for 5 years after study completion. All audio files will be destroyed when all the transcripts have been completed and verified. De-identified data will be stored digitally indefinitely as word documents which will be password protected.

***Approvals.*** The protocol, informed consent form, and participant information sheet were submitted to appropriate Research Ethics Committees in Oxford, Burkina Faso and Nigeria for approval. The investigators will submit and, where necessary, obtain approval from the above parties for all substantial amendments to the original approved documents.

***Informed consent.*** We designed an informed consent form to be administered to all prospective participants of the project after full disclosure of the study is read out to them from information sheets. Most of the interviews in Nigeria were conducted in English while those in Burkina were conducted in French. However, interviews with village health workers, parents and community members were conducted in local languages. Therefore, we translated the consent forms into the various local languages for the interviews with non-English speaking participants. 

***Discontinuation/withdrawal of participants from study.*** Each participant has the right to withdraw from the study at any time. In addition, the investigator may discontinue a participant from the study at any time if the investigator considers it necessary for any reason.

The audio recording will be deleted if the participant decides to withdraw mid-interview; the PIS for those participating in the FGDs states that if the participant withdraws mid-focus group, those portions of the audio recordings that capture his/her views will be deleted.

***Participant confidentiality.*** All interviewees have been assigned a unique identifier (an ID number) which will be used on all transcripts. Permission will be asked to mention the affiliation of the respondent in the results of the study. This is particularly important for national level representatives such as NMCP members and drug authorities with specific expertise related to their position. Real personal names and names of places of residence will not be included in any data. A database linking real names and ID numbers will be stored securely and separately from the data and will be accessible only to the research team.

***Data sharing and safety.*** Data collected for this study will be under the custodianship of MORU, will be de-identified and may be shared with other groups of researchers in accordance with the current data sharing policies. 

***Risks and benefits to research participation.*** The proposed project is considered a minimal risk study since it does not involve any invasive procedures. The study team does not expect any risks for participants beyond the minimal risks described above regarding confidentiality surrounding sensitive comments that might arise when participating in the qualitative interviews or group discussions. However, if there are any questions that the participant does not want to answer or discuss, these can be skipped and the interview or discussion can be stopped at any time.

***Benefits to research participation.*** There are no direct benefits to the participants. Their contribution to the project will inform the development of key policies and recommendations that will help identify the key ethical and market-related issues that should be taken into consideration when deploying triple artemisinin-based combination therapies in Africa.

***Compensation.*** We will follow the research practice at the various research sites and propose a token to be given to each participant in the interviews and FGDs. The proposed token will be discussed with the local ethics committee for approval before implementation.

A small token of appreciation will be provided in accordance with local customs (amount will be determined with local Ethics Committees). Snacks and drinks will be provided during interviews and FGDs where appropriate. Participants will be compensated for any actual transportation costs incurred to attend the interview or focus groups.

## Data availability

No data are associated with this article.
