# Supplementary material for: Ethical, Regulatory and Market related aspects of Deploying Triple Artemisinin-Based Combination Therapies for Malaria treatment in Africa: A study protocol
Source: Wellcome Open Res. 2021 Apr 7;6:75. [Version 1] doi: 10.12688/wellcomeopenres.16065.1 (PMC8378406; doi:10.12688/wellcomeopenres.16065.1)
Supplement: Peer review history

# Peer review: Reviewer response for version 1

**DOI:** 10.21956/wellcomeopenres.17622.r45245

**Published:** 2021-09-14

**Authors:** Jenny Hill

**Affiliations:** 1 Department of Clinical Sciences, Liverpool School of Tropical Medicine, Liverpool, UK

**Rationale and Objectives**

 This manuscript describes a protocol for a qualitative study to explore the views of key stakeholders on the key ethical, regulatory and market-related issues for the potential introduction of triple ACTs (TACTs) in the current scientific, economic and social contexts of two countries in Africa. Triple artemisinin-based combination therapies (TACTs), which combine existing co-formulated ACTs with a second partner drug that is slowly eliminated, may provide effective treatment and delay emergence of antimalarial drug resistance. The rationale for the study is the growing spread of resistance to ACTs from southeast Asia to Africa, for which there is increasing evidence in many parts of the continent.

 Specifically, the project aims to (i) examine the feasibility of deployment of TACTs, potential barriers to its deployment, to assess whether mitigation of observed barriers is indicated, and how the identified barriers can feed into communication plans; (ii) identify the key ethical, social, practical, and regulatory issues of changing first-line treatment from ACTs to TACTs; (iii) explore possible strategies to facilitate the integration of TACT in public and private sector distribution chains; (iv) explore the readiness of public and private sector markets for a transition to TACTs and (v) explore end-user acceptability of TACTs.

 One of the biggest barriers to the change from SP to ACTs for treatment of uncomplicated malaria in Africa was cost. Hence price, affordability and economic factors are important considerations that will need to be considered in any drug policy change and should be included in aim (ii). Another major consideration is the policy change process, policy drivers and policy triggers around ‘where’ and ‘when’ to deploy TACTs in terms of thresholds of ACT resistance. The authors should consider asking which other data that might be required by stakeholders in order to make policy decisions, who should collect these data, and which policy process or pathway countries will use to make the decision to introduce TACTs.

 The study is being conducted under the auspices of the DeTACT project (14 countries including 8 in Africa). Please include a link to the project for the benefit of readers. The authors’ justification for selecting the sites in Nigeria and Burkina Faso were due to the substantial burden of malaria in Nigeria (with 24% of malaria deaths globally), and the potential to prevent or delay artemisinin resistance at scale, and the different health systems and malaria policies between Nigeria and Burkina Faso. On searching WHO’s maps on drug resistance, I also noted that both countries have significant levels of the molecular marker for ACT resistance (Pfkelch13).

**Study design**

 The study design is appropriate and involves semi-structured interviews and focus group discussions with purposively selected key stakeholders including - representatives of national malaria control programs and regulatory agencies, public and private sector drug distributors and retailers, clinicians and prescribers, malaria researchers, parents, caregivers, and community leaders.

 If, as the authors state, this is the first study of its kind it would be advisable to include snowballing to allow inclusion of additional stakeholders identified as playing a relevant role during interviews held with the initial list of stakeholders identified. In addition, whilst the focus of the study is in two countries, it would be worth considering approaching regional bodies such as WHO-AFRO and networks involved in regulatory and ethical concerns in biomedicines, such as AVAREF. The AVAREF platform works to strengthen regulatory and ethics reviews, promote harmonized standards and approaches and accelerate the review of vaccines of high public health value among member countries, which calls for increased cooperation between donors, researchers, product developers, regulators and the medical ethics community.

 The inclusion/exclusion criteria are overly simplistic and are not reproducible as written (a stated objective for publishing the protocol). Greater clarity is needed e.g. a description of the roles of the people to be enrolled in each participant group or category. Presumably they would need to be senior level managers or decision-makers in MOH departments, regulatory agencies and other professional bodies.

 The authors state that the total number of interviews will depend on when data saturation is reached, however a target number or range (min-max) should be provided in the protocol. Similarly, the reasons for selecting different qualitative methods for different stakeholder groups should be explained. Note that while FGDs are useful for identifying ‘collective views’ they can also capture divergent views within homogeneous and heterogenous groups of participants.

 The study guides or the key themes/topics for each stakeholder group should be provided in tables or as supplementary files; using ‘etc.’ is not sufficient. A major barrier will likely include adherence to complex multiday regimens hence information from end-users about drug combinations or packaging and that inform drug information leaflets to promote adherence will likely be important, such as management of side effects, as well as affordability.

 There is inconsistency about the language to be used in the interviews between different sections of the protocol possibly in the authors’ attempt to describe what has been done so far (English in Nigeria) vs what is still planned (French in BF) – (See general comments at the end of this review). Clarity is needed on whether interviews conducted in French will first be transcribed in French before being translated to English (recommended).

 Data analysis needs to be described more clearly, in particular how the authors propose to approach coding of transcripts and whether any frameworks will be used for analysis for each of the different stakeholder groups, e.g. policy frameworks (policy makers) vs feasibility and acceptability frameworks (practitioners and end users). The authors state that the analysis will be based on the themes used to guide the interviews which are not available to the reader, nor how those themes were selected. If any frameworks are to be used as part of the analysis, these need to be added and referenced.

**General comments**

 As the study has commenced, the above suggestions to strengthen the study design may not be implementable in both countries though they may still be relevant for data collection in Burkina Faso.

 The protocol uses a mixture of future, present and past tense, presumably because the study is ongoing. For consistency, it might be prudent to stick with future tense in the protocol manuscript, rather than attempting to inform the readers about the study status, especially as no results are presented other than the number of participants recruited. This is however the editor’s decision.

 Key words should be expanded to include drug regulation/regulators, drug marketing, drug policy.

 The grammar needs checking, ‘the’ and plural(s) missing in places.

 Some of the background data and references needs updating e.g. most if not all countries in Africa have adopted ACTs for treatment of uncomplicated malaria, and there are more recent studies on drug resistance in Africa.

Is the study design appropriate for the research question?

Yes

Is the rationale for, and objectives of, the study clearly described?

Yes

Are sufficient details of the methods provided to allow replication by others?

Partly

Are the datasets clearly presented in a useable and accessible format?

Not applicable

Reviewer Expertise:

Implementation research, health systems and policy research, public health, mixed methods, evaluation

I confirm that I have read this submission and believe that I have an appropriate level of expertise to confirm that it is of an acceptable scientific standard.

# Peer review: Reviewer response for version 1

**DOI:** 10.21956/wellcomeopenres.17622.r45260

**Published:** 2021-08-19

**Authors:** James K. Tibenderana

**Affiliations:** 1 Malaria Consortium, London, UK; 2 London School of Hygiene and Tropical Medicine, London, UK

Summary

 The paper describes the protocol of an important study in readiness for the deployment of triple artemisinin combination therapies (TACTs) so that others have access to it and can attempt similar studies on the research questions. The study looks ahead at the ethical, social, regulatory and market position issues in readiness for the potential deployment of TACTs in Africa. The study comprises two main research questions on the ethical and market positioning around the deployment of TACTs. It is a qualitative study design using in-depth interviews and focus group discussions targeting key stakeholders in Burkina Faso and Nigeria.

Contribution, significance and novelty

 The protocol provides a useful opportunity for other researchers to feedback, and use to carry out similar studies.

Major concerns

 The study is very well written and clearly articulates the research questions, objectives and methodology. There are no major concerns regarding the paper

Minor concerns

 There are some minor issues that the authors could address in the paper to improve its clarity. They are as follows:

**Introduction**

 "recent reports suggest that these gains may stand the risk of being reversed, particularly with the threat of resistance to ACTs spreading from Southeast Asia to Africa": There are other threats such as insecticide resistance that are reversing the gains and good for the authors to mention so that the readers get a broad perspective on the potential threats. It is also worth noting that mechanisms of drug resistance to ACTs do include the chance of independent de novo mutations emerging in Africa.

**Aims and objectives**

 There are five study objectives and it is not clear how they align with the study design. The authors could mention in the methods how each of the objectives will be addressed. For example, it is not clear how the ethical issues are captured in the protocol to provide meaningful insights at the completion of the study, and yet they are very important as the authors have highlighted.

**Methods**

*Study participants*

 Do any of the key stakeholders represent the ethical (and social) issues regarding the deployment of TACTs? It will be useful to clarify this because it is unclear. The sample lists in Tables 1 and 2 do not provide information on respondents with possible insights into the ethical and social issues. Could this be clarified?

 The study respondents are to be sampled from different levels of the health system. It will be helpful to explain the sampling approach up to that level of the health system in particular since at the sub-national level the study population is larger and geographically dispersed. In the purposive sampling was maximum variation considered or not? The details will help with reproducibility, which is one of the reasons the authors give for publishing the protocol. In that regard, the authors could outline what measures were put in place to "ensure heterogeneity". I would also suggest that the parameters used to identify specific districts and study sites should be provided because the details will help guide other similar studies. Tables 1 & 2 provide the total number of interviews and focus group discussions by respondent and the authors say that the number will depend on when they reach data saturation; does this mean that the numbers in column 3 in the tables are indicative and could be exceeded?

 Information on the group of respondents that were used to pre-test the study tools will help to understand the reliability of the tools.

*Exclusion criteria*

 "not be recruited to the study" could be replace with "not invited to participate in the study". There is no mention in the methods how the authors plan to identify participants with intellectual and/or cognitive disability. Could this be provided in the methods?

**Data management**

 Information on the anticipated role of the sponsor in the study would be useful.

**Ethical and regulatory considerations**

*Discontinuation/withdrawal of participants from the study*

 It will be informative for researchers to know what possible reasons the investigators could decide to discontinue a participant from the study and still maintain the objectivity of the study. When it is left as a generic statement, it leaves the reader puzzled why the investigator/s would decide to discontinue any participant that has consented to participate in the study.

Summary recommendation

 The paper should be approved with minor revisions.

Is the study design appropriate for the research question?

Yes

Is the rationale for, and objectives of, the study clearly described?

Yes

Are sufficient details of the methods provided to allow replication by others?

Partly

Are the datasets clearly presented in a useable and accessible format?

Not applicable

Reviewer Expertise:

Epidemiology, implementation and operational research

I confirm that I have read this submission and believe that I have an appropriate level of expertise to confirm that it is of an acceptable scientific standard.

# Peer review: Reviewer response for version 1

**DOI:** 10.21956/wellcomeopenres.17622.r43978

**Published:** 2021-06-03

**Authors:** Eric Halsey

**Affiliations:** 1 Malaria Branch, US Centers for Disease Control and Prevention, Atlanta, GA, USA

This is a well-written protocol, with a clear rationale and appropriate study design. The background and methods are concise and well thought-out. The authors should be commended on having the foresight to seek information on downstream issues that may arise after the conclusion of this potentially paradigm-shifting study. I have no major criticism or points needing clarification.

Is the study design appropriate for the research question?

Yes

Is the rationale for, and objectives of, the study clearly described?

Yes

Are sufficient details of the methods provided to allow replication by others?

Yes

Are the datasets clearly presented in a useable and accessible format?

Not applicable

Reviewer Expertise:

Malaria and antimalarial efficacy

I confirm that I have read this submission and believe that I have an appropriate level of expertise to confirm that it is of an acceptable scientific standard.
